# Decoding PHR-Orchestrated Stress Adaptation: A Genome-Wide Integrative Analysis of Transcriptional Regulation Under Abiotic Stress in *Eucalyptus grandis*

**DOI:** 10.3390/ijms26072958

**Published:** 2025-03-25

**Authors:** Huiming Xu, Yifan Xing, Guangyou Li, Xin Wang, Xu Zhou, Zhaohua Lu, Liuyin Ma, Deming Yang

**Affiliations:** 1 Research Institute of Tropical Forestry, Chinese Academy of Forestry, Guangzhou 510520, China; seimei2020@163.com (H.X.); lgy@caf.ac.cn (G.L.); 18735788993@163.com (X.W.); luzhaohua@caf.ac.cn (Z.L.); 2 Center for Genomics, Fujian Provincial Key Laboratory of Haixia Applied Plant Systems Biology, School of Future Technology, College of Forestry, Fujian Agriculture and Forestry University, Fuzhou 350002, China; 13675008050@163.com (Y.X.); z18584014241@163.com (X.Z.)

**Keywords:** PHR, transcription factor, gene expression, cold stress, salt stress, phosphate starvation, nitrogen starvation, boron deficiency, *Eucalyptus grandis*

## Abstract

The phosphate starvation response (PHR) transcription factor family play central regulatory roles in nutrient signaling, but its relationship with other abiotic stress remains elusive. In the woody plant *Eucalyptus grandis*, we characterized 12 EgPHRs, which were phylogenetically divided into three groups, with group I exhibiting conserved structural features (e.g., unique motif composition and exon number). Notably, a protein–protein interaction network analysis revealed that EgPHR had a species-specific protein–protein interaction network: EgPHR6 interacted with SPX proteins of multiple species, while *Eucalyptus* and poplar PHR uniquely bound to TRARAC-kinesin ATPase, suggesting functional differences between woody and herbaceous plants. A promoter sequence analysis revealed a regulatory network of 59 transcription factors (TFs, e.g., BPC, MYBs, ERFs and WUS), mainly associated with tissue differentiation, abiotic stress, and hormonal responses that regulated *EgPHRs’* expression. Transcriptomics and RT-qPCR gene expression analyses showed that all *EgPHRs* dynamically responded to phosphate (Pi) starvation, with the expression of *EgPHR2* and *EgPHR6* exhibiting sustained induction, and were also regulated by salt, cold, jasmonic acid, and boron deficiency. Strikingly, nitrogen starvation suppressed most *EgPHRs*, highlighting crosstalk between nutrient signaling pathways. These findings revealed the multifaceted regulatory role of *EgPHRs* in adaptation to abiotic stresses and provided insights into their unique evolutionary and functional characteristics in woody plants.

## 1. Introduction

Phosphorus (P) is one of the main mineral nutrients required for plant growth and development [1,2]. Phosphorus is an important component of macromolecules such as adenosine triphosphate (ATP), phospholipids, DNA, and RNA and is required for phosphorylation [3,4]. Therefore, phosphorus is essential for many plant life processes such as energy metabolism, photosynthesis, respiration, cell membrane stabilization, and genetic information transmission [5,6]. The phosphorus that can be absorbed and utilized by plants is soluble inorganic phosphate (Pi), but Pi is easily fixed by cation such as iron and aluminum in the soil [7,8]. As a result, P has become the second largest environmental factor limiting plant growth and development after nitrogen in many ecosystems.

Plants have evolved complex low-Pi signaling systems that adapt to soil phosphate limitations through molecular and physiological regulatory mechanisms [6]. These processes include Root system architecture (RSA) remodeling and activation of phosphate transporter (PHT) expression to improve phosphate uptake efficiency, as well as the modulation of growth and development processes in response to phosphate availability [9]. Studies in model plants have shown that phosphate starvation responses (PHRs) are the core regulatory transcription factors of the plant systematic low-Pi signaling network [10]. Under low Pi conditions, cellular Pi and ATP levels are reduced in Arabidopsis and rice, inducing PPIP5K-mediated conversion of inositol polyphosphates (InsPs) from InsP7 to InsP6 instead of InsP8 [11]. As InsP8 can stabilize the SPXs–PHRs interaction and inhibit the release of PHR from its negative regulators, SPXs, the decrease in InsP8 levels at low Pi further destabilizes the PHRs–SPXs interaction, so PHRs can be freely released from the SPXs–PHRs interaction [12,13]. PHR then shuttles from the cytoplasm into the nucleus and activates the transcription of downstream phosphate starvation-inducing genes (PSIs) [12,13]. PHR binds to the conserved *cis*-elements P1BS (*GNATATNC*) in the promoter of the PSI gene and activates or inhibits gene transcription of ~3800 PSI genes in Arabidopsis [14]. These PHR-dependent PSI genes include phosphate transporters *PHT1;1* and *PHT1;4*, microRNAs *microRNA399* and *microRNA827*, and transcription factors such as *NIGT1* [15,16]. Conversely, PPIPK5 converts InsP7 to InsP8 and then stabilizes the SPXs–PHRs interaction under conditions of sufficient Pi [12,13]. As a result, the function of PHR is inhibited, and low-Pi signaling is disrupted [12,13]. Therefore, PHRs play an important role in regulating low-Pi signaling in plants. After the discovery of AtPHR1 in low-Pi signaling in 2001, a total of 15, 12, 20, 14, 18, 22, 23, 41, and 42 PHRs were characterized in Arabidopsis, rice, brachypodium, sorghum, *Zea mays*, *G. arboreum*, *G. raimondii*, *G. hirsutum* and *G. barbadense* [17,18,19]. Currently, 21 PHRs have been characterized in tea plants, and 2 PHRs from apple (MdPHR1) or 1 from poplar (PtoPHR1) have been functionally validated in response to low-Pi stress in woody plants [20,21,22]. However, research on PHR in many woody plants lags behind to a large extent compared to model plants and crops. In addition, research on PHR has focused on low-Pi signaling, and the function of PHR in other abiotic stresses remains largely unexplored.

*Eucalyptus* are perennial tall trees (rarely shrubs) from three genera: *Eucalyptus*, *Angophora*, and *Corymbia* in the family *Myrtaceae* of dicot plants, with a total of 1239 species (869 species and 370 subspecies), which are naturally distributed in Australia, Papua New Guinea, Indonesia, and the Philippines [23]. *Eucalyptus* is known for its rapid growth, with a maximum growth rate of ~10 m per year, superior wood performance, and strong environmental adaptability, resulting in *Eucalyptus* having been planted in more than 100 countries and regions, accounting for ~23% of the world’s forest plantations [24]. In China, *Eucalyptus* provides more than one-third of commercial wood production and is well adapted to the acidic soils of southern China, where effective free-Pi levels are known to be low due to the immobilization of free phosphorus by abundant cations such as aluminum and iron [7,8]. Therefore, understanding how *Eucalyptus* adapts and responds to low-Pi deficiency will be an interesting scientific question. However, to date, *PHR*s have not been characterized in *Eucalyptus*, and the expression patterns of *PHRs* under environmental stresses remain unexplored.

In this study, we performed a gene family analysis and characterized all 12 EgPHRs from *Eucalyptus grandis*, a model species in *Eucalyptus* spp. More importantly, we performed a systematic gene expression analysis of *EgPHRs* with different tissues, developmental stages, or environmental stresses. Our results provide useful preliminary results for further mechanistic studies of *EgPHRs*. It also unveils how *PHR* responds to different environmental stresses in woody plants and provides genetic information for the future design of high-phosphorus use efficiency (PUE) woody plants.

## 2. Results

### 2.1. Genome-Wide Characterization of PHRs in Eucalyptus grandis

A total of 12 PHR transcription factors in *Eucalyptus grandis* (*E. grandis*) were named from EgPHR1 to EgPHR12. The amino acid and CDS sequences of *EgPHRs* are listed in Table S1. Twenty-two PHR transcription factors were also characterized from *Populus trichocarpa* for the subsequent phylogenetic analysis (Table S1). To understand the biochemical properties of EgPHRs, we calculated the physicochemical properties of EgPHRs using Expasy (Table 1). In short, the amino acid length (<424.34 AAs) of all EgPHRs except EgPHR1 and EgPHR2 was shorter than the average length of plant proteins [25]. With the exception of EgPHR6, all EgPHRs showed higher isoelectric points (PI > 5.62) than the average PI of plant proteins [25]. As the instability index of all EgPHRs is above 40, the results suggested that EgPHRs might be unstable proteins [26]. All EgPHRs had a negative grand average of hydropathicity indicating that EgPHRs might be hydrophilic proteins [27]. Consistent with the results of many other plants, subcellular localization prediction showed that EgPHRs might be expressed in the nucleus.

### 2.2. Phylogenetic Analysis of Eucalyptus PHR Proteins

**Figure 1:**
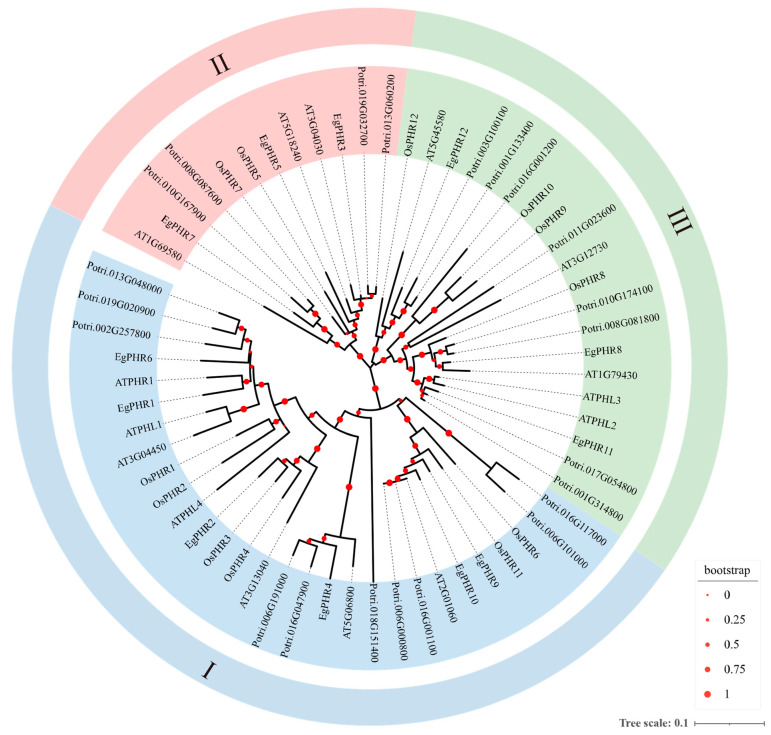
Phylogenetic analysis of the *PHR* gene family of *Eucalyptus grandis* with *Arabidopsis thaliana*, *Oryza sativa*, and *Populus trichocarpa*. Red dots indicate bootstrap values/metadata. The size of the circle corresponds to the level of bootstrap support for the branch. Circle diameters scale proportionally with bootstrap support values from 0 to 1, where larger diameters indicate stronger statistical confidence. Values exceeding 0.5 were generally considered reliable. The phylogenetic tree was constructed with MEGA7.0 using the maximum likelihood method with 1000 bootstraps. The tree uses three different colors to indicate the three evolutionary branches (**I**–**III**). The names of *PHRs* in *Arabidopsis*, *Oryza sativa*, *Populus trichocarpa*, and *Eucalyptus grandis* begin with “At”, “Os”, “Potri”, and “Eg”, respectively, at the beginning. The protein sequences of *Arabidopsis*, *Oryza sativa*, *Populus trichocarpa*, and *Eucalyptus grandis* PHRs are listed in Table S1.

To understand the evolutionary characteristics of EgPHRs, phylogenetic analyses were performed on protein sequences of 61 PHRs from *Arabidopsis thaliana* (15), rice (12), *Populus trichocarpa* (*Poplar*, 22), and *Eucalyptus* (12). The results showed that the PHR proteins were divided into three groups (groups I-III, Figure 1, Table S1). Group I was the most dominant clade (29 PHRs), and the most well-studied PHRs for Pi signaling regulation including AtPHR1, AtPHL1, OsPHR2, OsPHR3, and OsPHR4 belonged to that group [28,29,30]. Half of the *Eucalyptus* PHR proteins (EgPHR1, EgPHR2, EgPHR4, EgPHR6, EgPHR9, and EgPHR10) were clustered with six Arabidopsis, six rice, and ten poplar PHR proteins in group I (Figure 1). The number of group I PHRs was almost the same in all four species, suggesting that group I PHRs had a potentially conserved function in different species (Figure 1). Group II had the lowest number of PHR proteins (12 PHRs), with three EgPHRs (EgPHR3, EgPHR5, and EgPHR7), two Arabidopsis, two rice, and four poplar PHRs (Figure 1). Group III was the second largest clade (20 PHRs), but only three *Eucalyptus* PHRs (EgPHR8, EgPHR11, EgPHR12) were characterized, which was significantly lower than the PHR proteins of the other three species: five Arabidopsis, four rice, eight poplar PHR proteins (Figure 1).

### 2.3. Distinct Motifs Were Present or Absent Among Different Groups of PHRs

To further understand the function and conserved nature of EgPHR, a multi-sequence alignment was performed for the amino acid sequences of all 12 EgPHRs (Figure S1). Similar to PHRs from other plants, all EgPHRs contained two conserved domains: a Myb-like DNA-binding domain (Pfam ID: PF00249) and a Myb_CC_LHEQLE motif (Pfam ID: PF14379) (Figure S1). The results showed that EgPHR had a highly conserved PHR domain.

**Figure 2:**
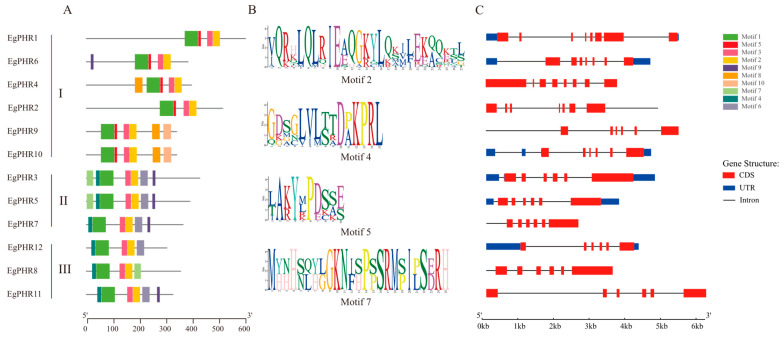
Conserved motifs and gene structure of the *Eucalyptus grandis* PHRs. (**A**) Conserved motifs of EgPHR; (**B**) sequence identification of several special EgPHR motifs; (**C**) genetic structure of *EgPHRs*, including CDS, UTR, and introns. The position of the sequence motifs, the domains, and the size of the exons or UTRs are estimated by the scale at the bottom.

To explore the function of EgPHR, the top ten enrichment motifs were characterized by MEMEs with all EgPHR amino acid sequences (Figure 2). Notably, all EgPHR genes had three conserved motifs: motif1, motif2, and motif3 (Figure 2A, Table S2). Two of them were the conserved Myb_DNA-binding domains (motif1) and Myb_CC_LHEQLE domains (motif3, Table S2). However, an additional motif (motif2, VQRHLQLRIEAQGKYLQKILEKAQKTL) was also ubiquitous in all EgPHR proteins. Interestingly, motif 4 (GDSGLVLTTDPKPRL) was absent in group I EgPHRs, but motif 5 (LAKYMPDSSE) was only present in group I EgPHRs. Motif 7 (MYNHSQYLGKNISPSSRMSIPSERH) occurred only in EgPHR3 and EgPHR5. As shown in Figure 2C, the gene structure analysis revealed that each group I EgPHRs had eight exons, while each group II or III EgPHRs contained only six exons (Table S3). Overall, the results suggested that group I EgPHRs might differ from the other two groups of EgPHRs in terms of conserved motifs and gene structure.

### 2.4. Gene Duplication and Syntenic Analyses of EgPHRs

**Figure 3:**
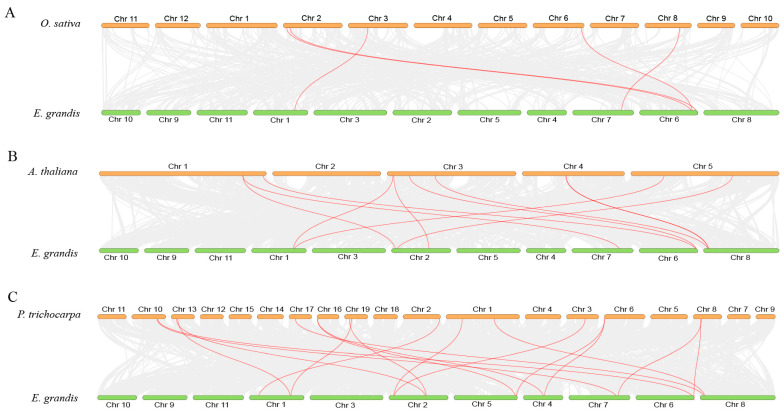
Analysis of covariance between *Eucalyptus grandis PHRs* and *Oryza sativa* (**A**), *Arabidopsis thaliana* (**B**), and *Populus trichocarpa* (**C**). Orange represents the chromosomes of *Eucalyptus grandis*, green represents the chromosomes of *Oryza sativa*, *Arabidopsis thaliana*, and *Populus trichocarpa* and the red line highlights *PHR* gene pairs with covariance.

To understand the origin of the *EgPHR* gene family, chromosomal distribution, gene duplication, and syntenic analyses of the *EgPHR*s were performed (Figure S2 and Figure 3). Gene duplication and syntenic analyses of *EgPHRs* showed that there were no tandem duplications within *EgPHRs*, while *EgPHR3* and *EgPHR5* were identified as a pair of segmental duplications (Figure S2). The syntenic analysis showed that *EgPHRs* formed orthologous gene pairs with ten Arabidopsis, five rice, and seventeen poplar *PHRs* (Figure 3, Table S4). The results suggested that *EgPHRs* were a highly conserved gene family, with the *EgPHRs* evolutionarily closer to poplar and Arabidopsis than rice *PHRs*.

### 2.5. The Potential Upstream Regulatory Transcription Factors of EgPHRs

**Figure 4:**
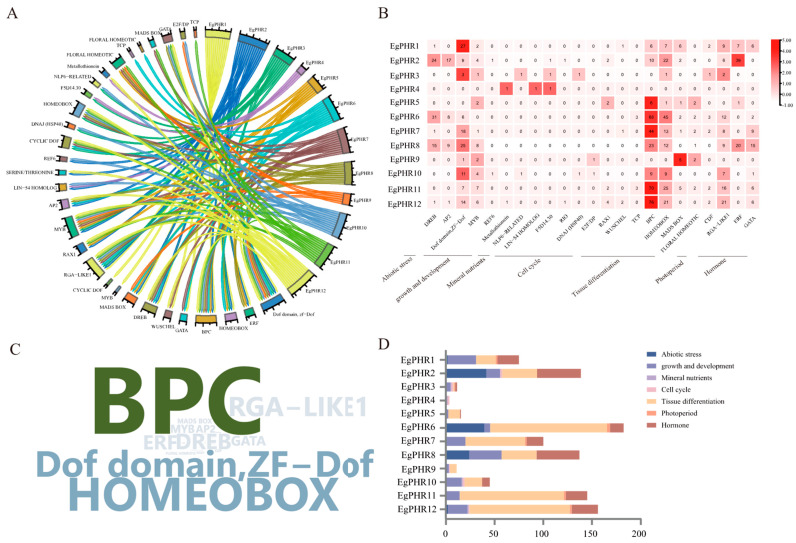
Prediction of potential regulatory transcription factors in the *Eucalyptus grandis PHR* promoters. (**A**) Network chords of predicted transcription factors targeting the *EgPHR* genes. The direction of the arrow is from *EgPHR*s to the transcription factors. (**B**) Statistics on the type of potential regulatory transcription factors in the promoter region of each gene. (**C**) The word cloud and font size of the transcription factors are positively correlated with the number of corresponding transcription factors. (**D**) Horizontal bar graph representing the number of transcription factors.

To further identify the potential transcription factors (TFs) that regulated gene expression in *EgPHRs*, the PlantRegMap database was introduced to characterize the regulatory transcription factors of *EgPHRs* (Figure 4, Table S5). A total of 59 transcription factors might regulate the gene expression of *EgPHRs*, and the potential transcription factors and *EgPHRs* formed a regulatory network chord (Figure 4A), and the binding sites of these TFs were located in the promoter region of *EgPHRs*. These 59 TFs were mainly composed of 9 MYBs, 4 ERFs (ERF035, LEP, ERF021, ERF003), 9 ZF-DoF (DOF2.4, DOF3.4, CDF2), 5 MADs-Box (SVP, AGL13), 5 homology boxes (SIP1, KNOX1, WUS, WOX13), 3 GATA (GATA1, GATA15, GATA24), 2 RAX1, 2 AP2, 3 DREB (DREB1A, DREB2A, DREB2F), 2 TCP (TCP18), 2 PBP (PBP6, PBP1), 1 REF (REF6), and 1 NLP (NLP6) (Figure 4B). Among all the TFs that regulated *EgPHRs*, BPC was the most abundant (312), followed by HOMEOBOX (156) and the Dof domain, ZF-Dof (121) (Figure 4B,C). *EgPHR6* was found to be the most transcriptionally regulated gene (182), followed by *EgPHR12* (156) and *EgPHR11* (145) (Figure 4B). Notably, *EgPHR12* and *EgPHR11* were predominantly regulated by BPC. Most of the BPC-binding motifs in the promoter region of the *EgPHR* genes had significant bias.

These 59 TFs could also be divided into seven categories, with tissue differentiation, abiotic stress, growth and development, and hormones being the top-four-abundance functional categories (Figure 4B,D). These results suggest that the expression of *EgPHRs* is highly regulated by transcription factors associated with tissue differentiation, abiotic stress, growth and development, and hormonal response.

### 2.6. Protein–Protein Interaction Network of EgPHRs

**Figure 5:**
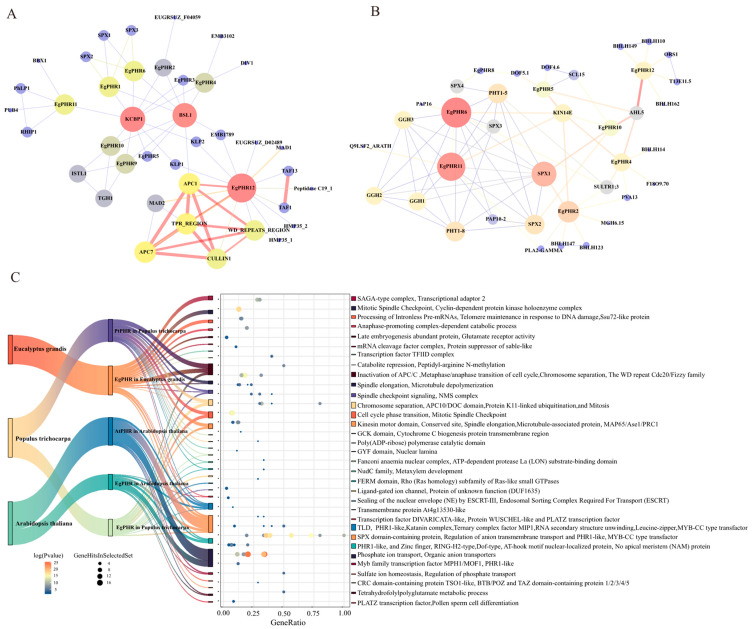
Protein–protein interaction network analysis and interaction protein enrichment analysis of PHR proteins in *Eucalyptus grandis*, *Populus trichocarpa*, and Arabidopsis. (**A**) Interaction network between EgPHR proteins and other *Eucalyptus grandis* proteins. (**B**) Interaction network between EgPHR proteins and Arabidopsis proteins. A network diagram consists of nodes and edges, each representing a protein; node-to-node connections (Edges) represent the interactions between these nodes; the connection’s line color, shading, and thickness indicate the degree of interaction, and the node color shading and node size indicate the thickness of the node (red: large, blue: small; thick: large, thin: small). (**C**) Cluster enrichment of EgPHR-, PtrPHR-, and AtPHR-interacting proteins in *Eucalyptus grandis*, *Populus trichocarpa*, and Arabidopsis.

To further elucidate the function of EgPHR, a protein–protein interaction (PPI) analysis of EgPHRs was performed with STRING (screening threshold: minimum required interaction score: medium confidence 0.4, maximum interaction shown: 30) (Figure 5A and Figure S3, Table S6). With the exception of EgPHR7 and EgPHR8, all EgPHRs had interacting proteins in *Eucalyptus*. SPXs were negative regulators of PHR and inhibited the Pi starvation response by interacting with and inhibiting the release of PHR [1]. EgPHR1 and EgPHR6 were the only two EgPHRs that interacted with EgSPXs in *Eucalyptus*. In addition to EgPHR1 and EgPHR6, EgPHR10 also interacted with PtrSPXs in poplars (Figure S3A, Table S6). However, only two EgPHRs, EgPHR11 and EgPHR6, interacted with AtSPXs in Arabidopsis (Figure 5B, Table S6). As a result, only EgPHR6 interacted with SPX proteins from all three species, and the protein–protein interaction network prediction showed that there were differences in the interaction between SPX and EgPHR in different species. PHT1 is a Pi transporter, and purple acid phosphatase (PAP) releases Pi from immobilized forms of P such as Al-P or Fe-P oxides [1]. Although no PHTs or PAPs interacted with EgPHRs in *Eucalyptus* from current data, EgPHR11 and EgPHR6 indeed interacted with PHT1 and PAP10-2 in Arabidopsis. The cluster enrichment analysis further supported the hypothesis that Pi signaling-related terms were enriched in EgPHR-interacting proteins from *Eucalyptus*, poplar, and Arabidopsis, and therefore, EgPHRs might play a role in the Pi starvation response.

Ten EgPHRs (EgPHR1, EgPHR2, EgPHR3, EgPHR4, EgPHR5, EgPHR6, EgPHR9, EgPHR10, EgPHR11, and EgPHR12) interacted with at least one of the TRAFAC myosin-kinesin ATPase superfamily proteins represented by BSL1, KCBP1, KLP1, and KLP2 in *Eucalyptus* (Figure 5A). The protein–protein interaction cluster enrichment analysis of *Eucalyptus* and poplar was enriched for mitotic spindle checkpoint signaling, positive regulation of chromosome segregation, spindle elongation, and microtubule depolymerization (Figure 5C, Table S7). Notably, PtrPHRs also interacted with the TRARAC-kinesin ATPase superfamily proteins in poplar (Figure 5C, Table S7), whereas EgPHR and AtPHRs did not interact with these proteins in Arabidopsis (Figure 5B and Figure S3C, Table S6). Therefore, PHR from two woody plants and PHR from Arabidopsis might have different functions.

### 2.7. Gene Expression Patterns of EgPHRs in the Development of Different Tissues

**Figure 6:**
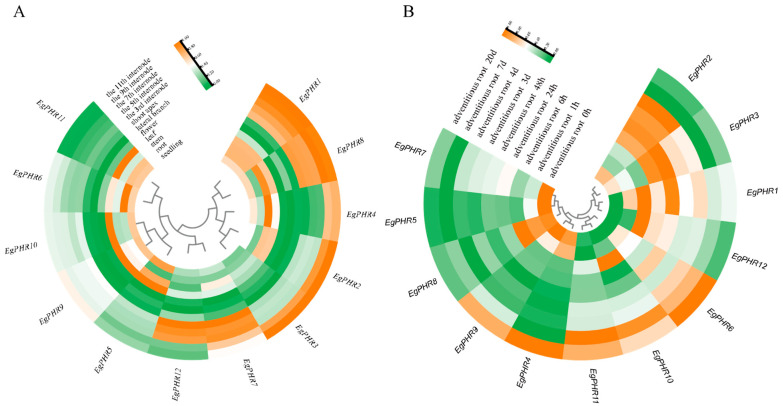
Gene expression analysis of *EgPHRs* in different *Eucalyptus* tissues and adventitious root development. Expression levels of *EgPHR*s in 12 different tissues (**A**) and 8 adventitious root induction states (**B**) in *Eucalyptus*. All samples were normalized using R software (v4.3.1) DEseq2 to obtain the final gene expression matrix. Heatmaps were plotted using R’s pheatmap and ggplot2 packages. The clustering is by rows, with orange representing high expression and green representing low expression.

To explore the potential role of *EgPHRs*, we analyzed the gene expression of *EgPHRs* in various tissues using transcriptome data from *Eucalyptus grandis* (Figure 6A, Table S8). The gene expression analysis showed that the expression of *EgPHRs* in different tissues such as seedlings, roots, stems, leaves, flowers, lateral branches, shoot apex, 3rd, 5th, 7th, 9th, and 11th internodes showed dynamic changes.

Generally, *EgPHR1* and *EgPHR8* clustered with each other and had a higher expression in nearly all tissues except for the flower, lateral branch, and shoot apex (Figure 6A). Notably, they were the only two *EgPHR*s that exhibited higher expression in all tested internodes. The slight difference between *EgPHR1* and *EgPHR8* was that *EgPHR8* showed a lower expression in the root but expressed higher in the stem (Figure 6A). The *EgPHR2*, *EgPHR3* and *EgPHR4* all showed a higher expression in the 9th and 11th internodes (Figure 6A). The difference among these three *EgPHR*s was that *EgPHR4* had the highest expression in the stem. *EgPHR7* and *EgPHR12* exhibited a higher expression in the 7th, 5th, and 3rd internodes. *EgPHR5*, *EgPHR9* and *EgPHR10* exhibited a relatively higher expression in the seedling and leaf. The slight difference was that *EgPHR5* also showed a relatively higher expression in the stem. *EgPHR6* and *EgPHR11* showed a relatively higher expression in the seedling, root and leaf.

From each tissue aspect, *EgPHR5* showed the highest expression, while *EgPHR3* had the lowest expression in seedlings (Figure 6A). In roots, *EgPHR6* had the highest expression level, and the other two *EgPHR*s (*EgPHR1* and *EgPHR11*) also showed a relatively higher expression level (Figure 6A). Taken together with the fact that *EgPHR6* and *EgPHR1* were predicted to interact with *EgSPX*s, we speculate that *EgPHR6* and *EgPHR1* might play central roles in the Pi deficiency signaling. Three *EgPHR*s (*EgPHR4*, *EgPHR5*, and *EgPHR8*) increased their expression in stem, suggesting that these *EgPHR*s might be involved in regulating stem development (Figure 6A). Many *EgPHR*s such as *EgPHR5*, *EgPHR9*, *EgPHR10*, and *EgPHR11* showed a higher expression in the leaf, indicating that *EgPHR*s might have important function in regulating leaf development (Figure 6A). All *EgPHR*s had a relatively lower expression in the lateral root and shoot apex, suggesting the *EgPHR*s might have limited roles in regulating the lateral root and shoot apex under normal conditions (Figure 6A). Seven *EgPHR*s (*EgPHR1*, *EgPHR2*, *EgPHR3*, *EgPHR4*, *EgPHR7*, *EgPHR8*, and *EgPHR12*) showed a higher expression in the development of different internodes. Overall, these results suggest that *EgPHR*s might play important roles in regulating the development of many *Eucalyptus* tissues.

### 2.8. Gene Expression Patterns of EgPHRs During the Adventitious Root Development

The proliferation of *Eucalyptus* depends on tissue culture, and the development of adventitious roots is one of the important characteristics of *Eucalyptus* production. To explore the potential function of *EgPHRs* in adventitious root (AR) development, we performed a gene expression analysis of *EgPHRs* using time-course (0 h, 1 h, 6 h, 24 h, 48 h, 3 days, 4 days, 7 days, and 20 days) transcriptome data during adventitious root development (Figure 6B, Table S9).

*EgPHR*s could be classified into three groups according to their gene expression pattern. The first group had five *EgPHR*s (*EgPHR4*, *EgPHR5*, *EgPHR7*, *EgPHR8*, and *EgPHR9*) (Figure 6B). They all had a higher expression in the control group, while showing a lower expression in many AR developmental stages (Figure 6B). There were several exceptions for these five *EgPHR*s. The first exception was *EgPHR8*, which had a higher expression at the 1 h and 6 h AR developmental stages (Figure 6B). The second exception was *EgPHR9*, which showed an increased expression at the 20-day AR developmental stage. The third exception was *EgPHR4*, which exhibited a higher expression at the 1 h and 20 days of AR developmental stage. The second group of *EgPHR* had three members (*EgPHR6*, *EgPHR10*, and *EgPHR11*) (Figure 6B). These three *EgPHR*s all had a higher expression at the 7-day and 20-day AR developmental stages, and with *EgPHR10* also having an increased expression at the 6 h AR developmental stage (Figure 6B). The last group of *EgPHR*s were *EgPHR1*, *EgPHR2*, *EgPHR3*, and *EgPHR12*. These four *EgPHR*s showed a higher expression at the 6 h (*EgPHR12*), 24 h (*EgPHR2* and *EgPHR3*), 48 h (*EgPHR1*, *EgPHR2*, and *EgPHR3*), 3-day (*EgPHR2*) and 4-day (*EgPHR2*) AR developmental stages (Figure 6B). Overall, these results suggest that *EgPHR*s might play an important role in AR development in *Eucalyptus grandis*.

### 2.9. Gene Expression Pattern of EgPHRs Under SA and JA Treatment

**Figure 7:**
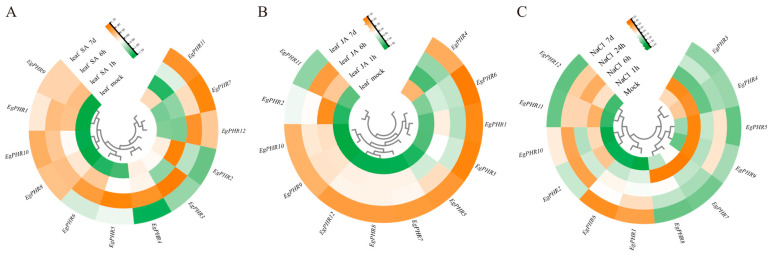
Gene expression analysis of *EgPHRs* under JA, SA, and salt stress treatments. The heatmap displays the expression levels of *EgPHRs* following treatment with SA (**A**) and JA (**B**) hormones, in the SA and JA treatment within 1 h, 6 h, and 7 h. (**C**) Expression levels of *EgPHRs* under salt stress, in the 200 mM NaCl treatment within 0 h, 1 h, 6 h, 24 h and 7d. All samples were normalized using R software DEseq2 to obtain the final gene expression matrix. Heatmaps were plotted using R’s pheatmap and ggplot2 packages. Clustering is by rows, orange represents high expression, and green represents low expression.

Pathogens threaten *Eucalyptus* growth and reduce the productivity of *Eucalyptus* plantations [31]. Two plant hormones, salicylic acid (SA) and jasmonic acid (JA), trigger plant immunity to defend against pathogen infection [31]. To understand whether *EgPHR*s were also involved in the regulation of *Eucalyptus* immunity, a gene expression analysis of *EgPHRs* in response to SA (leaf mock, 1 h, 6 h, and 7 d) and JA (leaf mock, 1 h, 6 h, and 7 d) was conducted with transcriptome data, respectively (Figure 7A, Table S10, Figure 7B, Table S11).

*EgPHR*s could be classified into eight groups according to their expression pattern under the SA treatment (Figure 7A). The first group contained four *EgPHRs* (*EgPHR1, EgPHR8, EgPHR9*, and *EgPHR10*), and they showed a higher expression under all SA treatment conditions (Figure 7A). The second group had *EgPHR5* and *EgPHR6*, and their expression was highly induced after 6 h of SA treatment (Figure 7A). The *EgPHR3* and *EgPHR4* constituted the fourth group, and they also had a higher expression after 6 h of SA treatment, while having a lower expression after 7 days of SA treatment (Figure 7A). *EgPHR2* was the only member in the fifth group, and it showed the highest expression after 1 h of SA treatment (Figure 7A). The sixth group only had *EgPHR12*, and its expression was induced after 6 h and 7 days of SA treatment. *EgPHR7* and *EgPHR11* were the only members in the seventh and eighth group, respectively (Figure 7A). They all showed a high expression after 7 days of SA treatment, while *EgPHR11* also showed a decreased expression after 1 day of SA treatment. Overall, these results suggest that all *EgPHR*s might be involved in response to SA in *Eucalyptus*.

Similarly, *EgPHR*s could be classified into four groups according to their expression pattern under the JA treatment (Figure 7B). The first group only had one member, *EgPHR11*, which showed the highest expression after 6 h of JA treatment. *EgPHR2* was the only member of the second group, and it showed the highest expression after 1 h of JA treatment (Figure 7B). The third group presented nine *EgPHR*s (*EgPHR1*, *EgPHR3*, *EgPHR5*, *EgPHR6*, *EgPHR7*, *EgPHR8*, *EgPHR9*, *EgPHR10*, and *EgPHR12*), and their expression was significantly induced after 7 days of JA treatment (Figure 7B). EgPHR4 was the only member of the last group, and it showed a higher expression under control and *EgPHR4* (Figure 7B). Overall, these results suggest that all *EgPHR*s might be involved in response to JA, especially under long-term JA treatment in *Eucalyptus*.

### 2.10. Gene Expression Pattern of EgPHRs Under Salt Stress

Salt stress is one of the major environmental stresses that limit plant growth and productivity [32,33]. To understand how *EgPHRs* respond to salt stress, the gene expression of *EgPHRs* was analyzed using transcriptome data in time-course salt-stress experiments (mock, 200 mM NaCl treatment for 1 h, 6 h, 24 h, and 7 days) (Figure 7C).

*EgPHR*s could be classified into four groups according to their expression pattern under salt stress. Four *EgPHR*s (*EgPHR2*, *EgPHR10*, *EgPHR11* and *EgPHR12*) showed a relatively higher expression after 1 h (*EgPHR2*, *EgPHR10*, *EgPHR11* and *EgPHR12*), 6 h (*EgPHR11* and *EgPHR12*), or 24 h (*EgPHR2*, *EgPHR10*, *EgPHR11* and *EgPHR12*) of salt stress (Figure 7C). The *EgPHR1* and *EgPHR6* together formed the second group; their expression was highly induced under 7 days of salt stress (Figure 7C). The third group comprised four *EgPHR*s (*EgPHR5*, *EgPHR7*, *EgPHR8* and *EgPHR9*), and they were highly induced under 1 h of salt stress (Figure 7C). The last group only contained *EgPHR3* and *EgPHR4*, and they were highly expressed under control and 1 h of salt stress (Figure 7C). Overall, these results suggest that all of *EgPHR*s might respond to salt stress at the transcriptional level in *Eucalyptus*.

### 2.11. Gene Expression Pattern of EgPHRs Under Cold Stress

**Figure 8:**
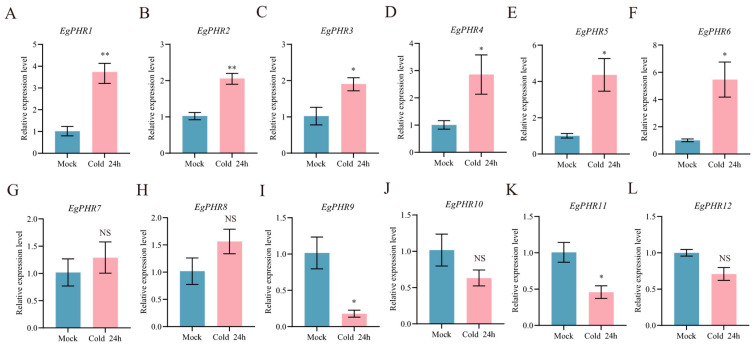
Gene expression analysis of *EgPHR*s in response to cold stress. (**A**–**L**) Expression of the *EgPHRs* in mock and cold treatment in the leaf. Seedlings were growth at 4 °C (cold stress) or 25 °C (control) for 24 h. *t*-test: “NS” represents no significance, “*” represents *p* < 0.05, “**” represents *p* < 0.01.

*Eucalyptus* plants are sensitive to cold damage, and cold stress is one of the main environmental stresses that permanently reduce the development and productivity of *Eucalyptus* plantations [34]. To test whether *EgPHRs* are involved in cold tolerance in *Eucalyptus*, gene expression analyses of *EgPHR*s were performed using RT-qPCR experiments with the leaves from seedlings under 24 h at 4 °C (cold stress) or 25 °C (control) conditions in *Eucalyptus grandis* (Figure 8A–L). Briefly, eight *EgPHR*s (*EgPHR1*, *EgPHR2*, *EgPHR3*, *EgPHR4*, *EgPHR5*, *EgPHR6*, *EgPHR9*, and *EgPHR11*) showed significant (*p* < 0.05) gene expression changes under cold stress (Figure 8A–F,I–K). Notably, cold stress significantly (*p* < 0.05) induced mRNA accumulation level of six *EgPHR*s (*EgPHR1*, *EgPHR2*, *EgPHR3*, *EgPHR4*, *EgPHR5*, and *EgPHR6*) (Figure 8A–F). Conversely, two *EgPHR*s, *EgPHR9* and *EgPHR11*, significantly (*p* < 0.05) reduced the mRNA accumulation level under salt stress (Figure 8I,K). Therefore, cold stress indeed affected the gene expression of many *EgPHRs* at the transcriptional level.

### 2.12. Gene Expression Pattern of EgPHRs Under Low-Phosphate Starvation

To investigate the role of *EgPHR*s in response to Pi starvation, a gene expression analysis of *EgPHRs* was performed using RT-qPCR experiments using the roots under different time-courses of Pi starvation (control: 0.5 mM KH_2_PO_4_; Pi starvation, LP: 0.005 mM KH_2_PO_4_ treatment for 6 h, 12 h, 24 h, and 3 days).

**Figure 9:**
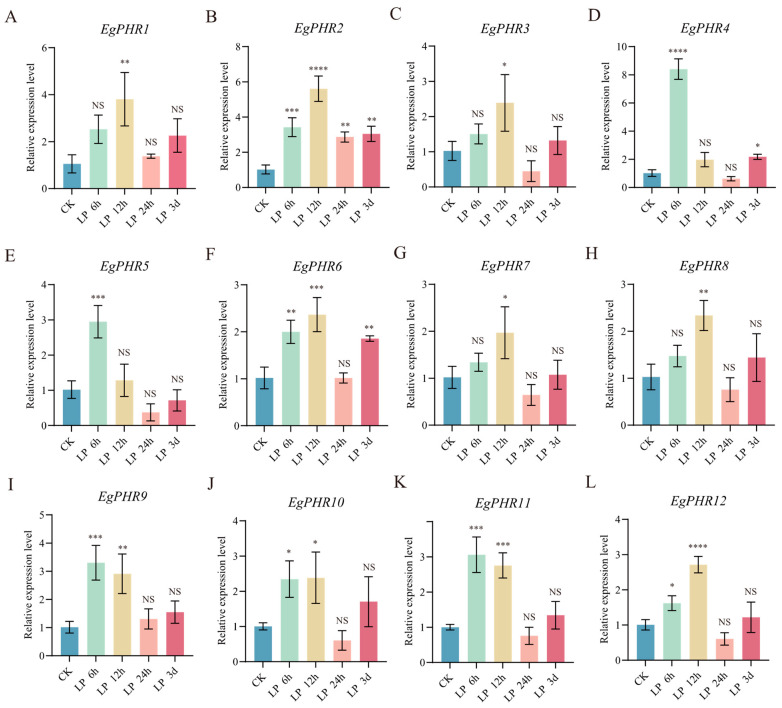
Gene expression analysis of *EgPHR*s in response to phosphate deficiency. (**A**–**L**) General overview of the expression of the *EgPHRs* in phosphate deficiency treatment in CK, 6 h, 12 h, 24 h and 3 d in roots. Two-way ANOVA test: “NS” represents no significance, “*” represents *p* < 0.05, “**” represents *p* < 0.01, “***” represents *p* < 0.001, “****” *p* < 0.0001.

Generally, Pi starvation induced the expression of all *EgPHR*s in at least one LP condition (Figure 9A–L). After 6 h of LP condition, Pi starvation significantly (*p* < 0.05) induced the expression of eight *EgPHR*s (*EgPHR2*, *EgPHR4*, *EgPHR5*, *EgPHR6*, *EgPHR9*, *EgPHR10*, *EgPHR11*, *EgPHR12*) (Figure 9B,D,E,F,I–L), and five *EgPHR*s (*EgPHR2*, *EgPHR4*, *EgPHR5*, *EgPHR9*, and *EgPHR11*) dramatically increased their expression (*p* < 0.001) (Figure 9B,D,E,J,L). Similarly, LP induced most *EgPHR*s’ (nine *EgPHR*s: *EgPHR1*, *EgPHR2*, *EgPHR6*, *EgPHR7*, *EgPHR8*, *EgPHR9*, *EgPHR10*, *EgPHR11*, and *EgPHR12*) expression under a 12 h LP treatment conditions (Figure 9A,B,F–L). Among them, four *EgPHR*s (*EgPHR2*, *EgPHR6*, *EgPHR11*, and *EgPHR12*) had dramatically (*p* < 0.001) increased gene expression after 12 h of LP treatment (Figure 9B,F,K,L). Interestingly, *EgPHR2* was the only *EgPHR* that increased its expression under a 24 h LP treatment. Similarly, LP induced the expression of only three *EgPHR*s (*EgPHR2*, *EgPHR4* and *EgPHR6*) after 3 days of LP treatment (Figure 9B,D,F). Notably, *EgPHR2* was the only *EgPHR* that was induced by LP under all treatment conditions, and *EgPHR6* was the next *EgPHR* that was regulated by LP under three of the four LP treatment conditions (Figure 9). Overall, these results suggest *EgPHRs* indeed play a vital role in coping with Pi starvation.

### 2.13. Gene Expression Pattern of EgPHRs Under Nitrogen Starvation

**Figure 10:**
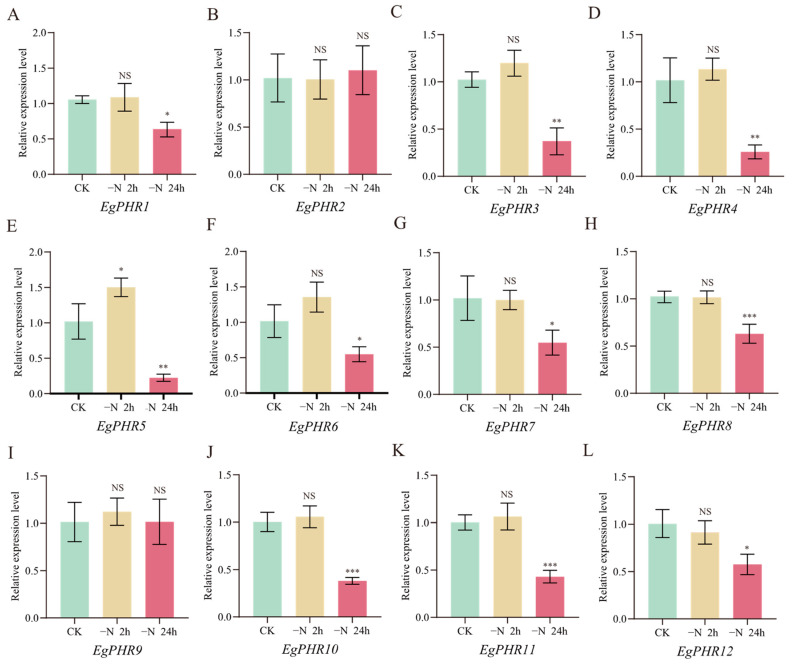
Gene expression analysis of *EgPHRs* in response to nitrogen starvation. (**A**–**L**) General overview of the expression of the *EgPHRs* in CK, 2 h, and 24 h low nitrogen treatment in roots. CK: 10 mM KNO_3_ treatment, −N: 0 mM KNO_3_ treatment. One-way ANOVA test: “NS” represents no significance, “*” represents *p* < 0.05, “**” represents *p* < 0.01, “***” represents *p* < 0.001.

Nitrogen and phosphate interact with each other, and the addition of nitrate can partially rescue the Pi starvation phenotype [1,35]. To understand whether nitrogen starvation also affects the Pi starvation response, the gene expression of *EgPHRs* was analyzed by RT-qPCR using the root tissues under nitrogen starvation conditions (control: 10 mM KNO_3_, nitrogen starvation, −N: 0 mM KNO_3_ for 2 h and 24 h). Generally, nitrogen starvation significantly affected the gene expression of all *EgPHR*s except *EgPHR2* and *EgPHR9* (Figure 10). Nitrogen starvation only significantly (*p* < 0.05) induced the gene expression of *EgPHR5* but not other *EgPHR*s after 2 h of nitrogen starvation (Figure 10E). Conversely, nitrogen starvation significantly (*p* < 0.05) repressed the expression of all *EgPHR*s except *EgPHR*2 and *EgPHR9* after 24 h of nitrogen starvation (Figure 10). Overall, nitrogen starvation might negatively regulate the gene expression of *EgPHR*s at the transcriptional level.

### 2.14. Gene Expression Pattern of EgPHRs Under Boron Deficiency

Boron deficiency greatly reduces the forest productivity by repressing the development of the shoot apex, leading to the top dieback and leaf chlorosis [36]. Boron (B) and P might interact with each other. To test whether B could affect the Pi signaling, the gene expression of *EgPHRs* was assessed via time series experiments in *Eucalyptus* (leaves: mock, 6 h, 24 h, 2 days, 4 days, and 21 days; roots: mock, 6 h, 24 h, 2 days, 4 days, and 21 days) (Figure S4, Table S12).

Generally, *EgPHR*s could be classified into six groups according to their expression pattern under B deficiency. The first group only contained *EgPHR10*, which showed the highest expression in leaves under a 6 h B deficiency treatment (Figure S4). *EgPHR6* was the only member of the second group and showed the lowest expression in roots after 24 h, 2 days, and 4 days) (Figure S4). The third group had seven *EgPHR*s (*EgPHR1*, *EgPHR2*, *EgPHR3*, *EgPHR4*, *EgPHR5*, *EgPHR7*, and *EgPHR9*), and they showed a relative higher expression in leaves after 6 h, 24 h, 2 days, 4 days, or 21 days of B deficiency treatment (Figure S4). *EgPHR12* was the only member in the fourth group and increased its expression in roots under the B deficiency treatment (Figure S4). The fifth group only contained *EgPHR11*, which showed the highest expression in roots under control and decreased expression in roots under B deficiency treatment (Figure S4). *EgPHR8* was the only member of the last group. Its expression was suppressed in leaves after 6 h, 24 h, and 2 days of B deficiency treatment, which increased its expression in roots after 2 days and 21 days of B deficiency treatment (Figure S4). Overall, these results suggest that boron deficiency might also regulate the gene expression of *EgPHR*s at the transcriptional level.

## 3. Discussion

Sessile plants are subject to many environmental stresses throughout their life cycle, including nutrient deficiencies [37,38]. As a result, plants must coordinate their complex growth with nutrient availability. Phosphorus is an essential macronutrient for all eukaryotes, but how woody plants adapt to phosphorus deficiency remains largely unclear [1]. *Eucalyptus* is one group of the important hardwoods, and is an important source of wood, furniture, and pulp worldwide due to its fast growth rate, short rotation period (*Eucalyptus* plantations are typically managed under short rotation cycles of 5–7 years to maximize biomass yield through rapid growth and frequent harvesting in intensive silvicultural systems), and good wood properties [39]. Therefore, characterization of PHR transcription factors will provide important genetic information for engineered high-phosphate use efficiency (PUE) *Eucalyptus* plants.

A total of twelve EgPHRs were characterized in *Eucalyptus grandis*. The number of EgPHRs were dramatically reduced compared to PHRs from the grass species (14~42 *PHRs* in Arabidopsis, brachypodium, sorghum, *Zea mays*, *G. arboreum*, *G. raimondii*, *G. hirsutum*, and *G. barbadense*) and the woody plant species (21~22 in tea plants and poplar) (Table 1 and Table S1) [17,18,19,20]. The only exception was rice PHRs (12 PHRs) [17]. It would be very interesting to analyze further in the future why fast-growing species have few PHRs, and the implication of that on high-PUE efficiency.

Phylogenetic analyses from *Eucalyptus*, Arabidopsis, rice, and poplar revealed that PHR proteins could be classified into three groups. The group I PHRs had the most well-studied PHRs that respond to Pi deficiency from model plants, including AtPHR1, AtPHL1, and OsPHR2 (Figure 1) [1,28]. Six *Eucalyptus* PHRs including EgPHR1, EgPHR2, EgPHR4, EgPHR6, EgPHR9, and EgPHR10 belonged to group I PHRs (Figure 1). Taken together with the fact that EgPHR1 and EgPHR6 interacted with SPXs in the protein–protein interaction analysis and the expression of *EgPHR1*, *EgPHR2*, *EgPHR6*, and *EgPHR10* were induced under Pi deficiency (Figure 6A), we then speculated that group I PHRs might also be involved in regulating Pi signaling in *Eucalyptus*. Notably, all group I EgPHRs had a unique motif (LAKYMPDSSE) (Figure 2B, Table S2), and it will be interesting to test in the future whether this motif play an important function on the regulation of EgPHR-mediated Pi signaling.

Group II PHRs comprised the smallest subset, with three EgPHR members (EgPHR3, EgPHR5, and EgPHR7) belonging to this category (Figure 1). Functional characterization has been reported for two genes within that group. The GARP-type transcription factor MYR2 (At3G04030) negatively regulates nitrogen reutilization in Arabidopsis by suppressing asparagine synthetase 1 (ASN1) expression, thereby delaying nutrient starvation- and dark-induced leaf senescence under light–dark cycles in vascular tissues [40]. MYR1 (At5G18240) and MYR2 are associated with light intensity responses, repressing flowering and organ elongation under low-light conditions through the inhibition of GA20ox2 expression, which modulates bioactive gibberellin levels [41]. These findings suggest that group II EgPHRs may also play important roles in light-regulated nitrogen reutilization and organ elongation processes.

The Arabidopsis PHR1 family members form two distinct regulatory modules: PHR1/PHL1 primarily drives core phosphate starvation responses (PSR) by activating phosphate uptake genes and maintaining phosphorus homeostasis under Pi deficiency, while PHL2/PHL3 modulates growth regulation under normal conditions and fine-tunes stress adaptation through synergistic or antagonistic interactions with PHR1/PHL1 [19]. Structural divergence underpins their functional specialization—PHR1/PHL1 dimerizes via N-terminal extensions to activate P1BS-dependent PSR pathways, whereas PHL2/PHL3’s unique C-terminal domains restrict interactions to their own cluster, enabling the coordinated regulation of secondary metabolism and chromatin remodeling [19]. Given that *Eucalyptus grandis*’s Group III PHRs (EgPHR8, EgPHR11, and EgPHR12) share phylogenetic clustering with Arabidopsis PHL1/PHL3 (Figure 1), these *EgPHRs* may balance growth–stress trade-offs through structural adaptations in N-/C-terminal domains, potentially mediating species-specific PSR strategies in woody plants.

Phosphate and nitrate are two of the most important mineral nutrients for plant development and productivity [1]. More importantly, a proper N:P ratio is critical for plant growth [42]. Previous studies from Arabidopsis and rice have suggested that plants adopt a NRT1.1-SPX module to simultaneously regulate the nitrate and phosphate deficiency signaling [43]. Briefly, OsNRT1.1B interacts with OsSPX4, and OsSPX4 is the negative regulator for OsPHR2 and OsNLP3 [43]. Similarly, a study in Arabidopsis proved that ~85% of phosphate starvation response genes had their expression dependent on nitrate [35]. Therefore, nitrate regulates the expression of phosphate starvation responses genes. In this study, low nitrogen availability significantly suppressed the expression of nine *EgPHRs* (including *EgPHR6* and *EgPHR10*) in roots (Figure 10), demonstrating that nitrogen promotes phosphorus-related responses in *Eucalyptus*, and the repression of PHR expression under nitrogen deficiency aligns with observations in other species such as Arabidopsis. Notably, *EgPHR6* and *EgPHR10* were also induced by Pi deficiency (Figure 9). Therefore, nitrogen might interact with phosphate starvation responses in *Eucalyptus*. The hypothesis were further supported by an observation that nitrate-associated transcription factor NLP6’s binding sites were characterized in the promoter of *EgPHR10* (Figure 4, Table S5). This result further proves that N and P might interact with each other in *Eucalyptus*. A future functional analysis might be performed to further prove whether the EgNLP6-EgPHRs module might regulate the *Eucalyptus* N–P balance.

The interaction between boron (B) and phosphate (P) exhibits both antagonistic and synergistic relationships depending on plant species and environmental conditions [44,45,46,47]. Notably, in this study, B deficiency suppressed the expression of *EgPHRs* in roots, potentially due to disrupted P transport systems or competitive absorption dynamics (Figure S4). However, B paradoxically upregulated certain *EgPHRs* homologs in leaves, suggesting a compensatory adaptation to maintain P homeostasis under B deficiency (Figure S4). This dual regulation aligns with prior findings: B and P interactions involve shared transport pathways (e.g., competitive inhibition in tomato and maize), while moderate P availability enhances B uptake by improving rhizosphere conditions and transpiration-driven absorption (e.g., in *Brassica napus*) [45,46,47]. Extreme B deficiency or toxicity exacerbates P imbalance, as seen in *Vicia faba* and groundnuts, whereas excessive P fertilization reduces B accumulation [44], highlighting their tightly coupled yet antagonistic roles in nutrient dynamics. The observed tissue-specific *EgPHR* expression in *Eucalyptus grandis* under B deficiency may reflect evolutionary strategies to optimize P utilization under fluctuating nutrient stresses, consistent with the conserved PHR-mediated crosstalk between environmental cues and nutrient signaling.

The interplay between jasmonate (JA) and phosphate signaling converges on phosphate starvation response’s (PHR) transcription factors as evolutionary conserved regulatory hubs. In this study, prolonged exogenous JA treatment induced the upregulation of 11 *EgPHRs* in *Eucalyptus grandis* (Figure 7B), likely reflecting an adaptive strategy to enhance P acquisition/utilization efficiency under JA-mediated stress responses. This aligns with mechanistic insights from other species: Arabidopsis PHR1 coordinates JA signaling by interacting with JAZ repressors and MYC2 to activate JA-responsive genes (e.g., anthocyanin biosynthesis), while tea plant’s CsPHRs integrate P signaling with JA pathways through JAZ degradation-triggered activation of the secondary metabolism [48,49]. However, functional specialization among the 11 *EgPHRs* in *Eucalyptus* requires further investigation to clarify their roles in balancing JA-mediated defense and P homeostasis.

Phosphate represses plant height in grass including Arabidopsis and rice as well as woody plants such as poplar and Chinese fir [1,50,51,52]. However, the molecular mechanism of how Pi regulates cell cycle and shoot apex remain unclear. In particular, it is still not known whether the functions between woody and grass PHRs are different from each other. In this study, the promoter analysis and protein–protein analysis both showed that *Eucalyptus* PHRs might have different functions compared to grass PHRs (Figure 4 and Figure 5). The promoter analysis revealed that tissue differentiation and cell-cycle-related transcription factors such as WUS, KNOX1, WOX13, and WUS had binding sites in the promoters of *EgPHRs* (Figure 5C). WUS is the critical regulator that modulates the cell fate determination in plants [53,54]. KNOX1 is an important transcription factor in tissue differentiation and cell cycle, and the loss of KNOX1 leads to failure of the Shoot Apical Meristem (SAM) establishment [55]. KNOX1 also functions on the generation of diploid plants [55]. WOX13 negatively regulates the SAM formation of WUS [56]. SIP1 has been reported to be upregulated in spindle cell carcinoma of the head and neck in human [57]. RAX1 is required for the diploid bipolar budding pattern in budding yeast [58]. Therefore, tissue differentiation and cell-cycle-related transcription factors might regulate the gene expression of *EgPHRs*. BSL1 regulates cell fate asymmetry and leads the mother cell to produce daughter cells with different fate during mitosis [59]. KCBPs play important roles in the spindle microtubule convergence in anaphase to ensure that each sister chromosome group has coherence kinetochore-fibers [60]. In this study, the protein–protein interaction analysis unveiled that all EgPHRs except EgPHR7 and EgPHR8 could interact with TRAFAC class myosin-kinesin ATPase superfamily proteins such as BSL1, KCBP1, KLP1, and KLP2 in *Eucalyptus* (Figure 5A). Similar observation was also identified in the protein–protein interaction analysis of poplar PHRs (Figure S3B). In addition, EgPHRs also interacted with DIVARICATA-like and WUSCHEL-like proteins (Figure 5C). Therefore, we speculated that PHRs might also be involved in tissue differentiation and cell-cycle regulation in woody plants. However, these results originated from either cis-elements’ screening or interacting based on the protein’s amino acid sequences. Functional analyses will be required to further prove the hypothesis that woody plant PHRs might differ from grass PHRs and modulate the cell cycle and tissue differentiation. Overall, our results suggest that *EgPHRs* may play an important role in development, abiotic stress tolerance, and mineral nutrient starvation adaptation.

## 4. Materials and Methods

### 4.1. Characterization of EgPHRs in Eucalyptus grandis

The *Eucalyptus grandis* genomic dataset utilized in this investigation was acquired from the NCBI Sequence Read Archive under the whole-genome sequencing (WGS) project JABKBJ01. For the systematic identification of PHR family proteins in *Eucalyptus grandis*, two specialized Hidden Markov Model (HMM) profiles—Myb_CC_LHEQLE (PF14379.9) and Myb_DNA-binding (PF00249.34)—were retrieved from the Pfam protein family database (http://pfam-legacy.xfam.org/) (accessed on 21 August 2024). These conserved domain profiles were subsequently employed in HMMER software (v3.0) to perform comprehensive a genome-wide screening and characterization of potential EgPHR candidates through sequence alignment and domain architecture analysis [61].

The candidate EgPHR proteins were then submitted to three different databases: NCBI-Batch CD-search (https://www.ncbi.nlm.nih.gov/Structure/bwrpsb/bwrpsb.cgi) (accessed on 21 August 2024), Pfam (http://pfam-legacy.xfam.org/) (accessed on 21 August 2024), and SMART (https://smart.embl.de/) (accessed on 21 August 2024) to verify its conserved domain. Proteins with all two conserved domains (Myb_CC_LHEQLE and Myb_DNA binding) were considered as EgPHR proteins. Subsequently, comprehensive physicochemical characterization was performed via ExPASy ProtParam (https://web.expasy.org/protparam/) (accessed on 21 August 2024), quantifying critical biochemical parameters including amino acid composition, molecular mass (kDa), theoretical isoelectric point (pI), grand average of hydropathicity (GRAVY), and instability index to assess structural stability [61]. Subcellular localization was predicted by Plant-mPLoc (http://www.csbio.sjtu.edu.cn/bioinf/plant-multi/#) (accessed on 21 August 2024).

### 4.2. Phylogenetic Analysis

The PHR amino acid sequences of Arabidopsis were downloaded from TAIR (https://www.arabidopsis.org/) (accessed on 22 August 2024) and the amino acid sequences of *Populus trichocarpa* and *Oryza sativa* were downloaded from the Phytozome database (https://phytozome-next.jgi.doe.gov/) (accessed on 22 August 2024), respectively. Pairwise sequence comparison of these three species with *Eucalyptus grandis* PHR protein was performed using MEGA7.0. After a comparison using Quick Run TrimAL tool from TBtools (v2.154) [62], the sequence was pruned to achieve maximum likelihood evolutionary tree construction (1000 replicates of a bootstrap analysis) and the evolutionary tree was beautified on the ITOL website (https://itol.embl.de/) (accessed on 23 August 2024).

### 4.3. Conserved Domains, Motifs, and Gene Structure Analysis

A multi-sequence comparison of EgPHR proteins was performed using DNAman8 software [63]. We uploaded the EgPHR protein sequences to the MEME webpage—MEME Suite (https://meme-suite.org/meme/) (accessed on 24 August 2024), set the number of patterns we wanted to discover to 10, and clicked the MAST XML output to download the results. We then extracted the annotation information of *EgPHRs* and visualized the gene structure using the GSDS website (https://gsds.gao-lab.org/index.php) (accessed on 24 August 2024).

### 4.4. Duplication Event Analysis and Multi-Species Analysis of Covariance

A self-comparison of protein sequences from *Eucalyptus grandis* were conducted using the Blast Compare Two Seqs module from TBtools [62]. We used Quick run MCScanX Wrapper (a built-in tool for TBtools) to obtain a link file of the relationships between genes and visualized them with the Circle Gene View. A multi-species analysis of the covariance between rice, Arabidopsis, *Populus trichoria*, and *Eucalyptus* was conducted and visualized using one-step MCScanX-Ultrafast and Dual System Diagram for McscanX tool from TBtools [62].

### 4.5. Prediction and Analysis of Potential Upstream Regulatory Transcription Factors for EgPHRs

The promoter sequence 2 kb upstream of the *EgPHR* genes was extracted and submitted to the PlantRegMap database (https://plantregmap.gao-lab.org/regulation_prediction.php) (accessed on 3 December 2024) to predict the upstream regulatory transcription factors. We used Origin2024 to draw chord diagrams and TBtools Heatmap tools for classification and heatmap drawing. We plotted word clouds and bar stacks using the ggplot2 package in R [64].

### 4.6. Protein–Protein Interaction Analysis

*Eucalyptus grandis* PHR protein sequences were submitted to the STRING website (https://cn.string-db.org/) (accessed on 13 September 2024), and *Eucalyptus grandis*, *Populus trichocarpa*, and *Arabidopsis thaliana* were selected as the reference species to predict the interaction network between EgPHR, PtrPHR, and AtPHR proteins. The detailed parameters included the full STRING network, evidence in meaning of edges, medium confidence (0.400) in minimum required interaction score, and no more than 30 interactors in the max number of interactors to show. TSV result files in string_interactions_short format were downloaded to organize and beautify protein interaction networks using Cytospace (version 3.6.1). All enriched terms through the Analysis module were downloaded, the bubble diagram was plotted using the ggplot2 package in R, and the sankey diagram was plotted using Origin2024.

### 4.7. Abiotic Stress Treatments and RT-qPCR Analysis

*Eucalyptus grandis* seeds were harvested in May 2024 from 15-year-old *Eucalyptus grandis* trees at the Baisha State-owned Forest Farm in Minhou, Fujian Province, and stored in a −20 °C freezer. *Eucalyptus grandis* seeds were sown on vermiculite and germinated and grown in a greenhouse with an ambient temperature of 25 °C, 16 h of light/8 h of darkness, with a light intensity of 50 μmol, and a humidity of 60%. *Eucalyptus* seedlings that were 1.5 months old and growing consistently were selected and grown in 1/2 Hoagland nutrient solution (2.5 mM KNO_3_, 0.5 mM KH_2_PO_4_, 1 mM MgSO_4_, 2.5 mM Ca(NO_3_)_2_·4H_2_O, pH 5.0) for three weeks. *Eucalyptus* seedlings were divided into three groups: control group (0.5 mM KH_2_PO_4_, 10 mM KNO_3_, hereinafter referred to as CK), nitrogen starvation group (0 mM KNO_3,_ hereinafter referred to as -N) and LP group (0.005 mM KH_2_PO_4_, hereinafter referred to as LP) for hydroponic treatment. Root sampling was performed at 2 h and 24 h for the -N treatment, and root sampling was performed at 6 h, 12 h, 24 h, and 3 days for the LP treatment.

The 1.5-month-old seedlings of *Eucalyptus grandis* (watered once a week with 2 L 1/2 Hoagland nutrient solution, pH 5.0) were grown in a 4 °C low-temperature incubator or at 25 °C with identical light and humidity condition, and the leaves were sampled after 24 h. Total RNA extraction from roots and leaves of *Eucalyptus grandis* were performed using the RNAprep Pure Plant Plus Kit (Polysaccharides & Polyphenolics-rich) (TIANGEN, Beijing, China). Total RNAs were reverse-transcribed using the Evo M-MLV Reverse Transcription Premixed Kit with gDNA removal (Accurate Biotechnology, Changsha, China). RT-qPCR reactions of 12 *EgPHR* genes were performed on a QuantStudio 1 Plus Real-Time PCR System (Thermo Fisher Scientfic, Waltham, MA, USA) using the SYBR Green Pro Taq HS Premixed qPCR Kit (with Rox) (Accurate Biotechnology, Changsha, China). Primers used in this study are listed in Table S13. The RT-qPCR procedure was as follows: 95 °C for 30 s, followed by 40 cycles of 95 °C for 5 s and 58 °C for 30 s, followed by a final step of 72 °C for 30 s. The *ACTIN1* gene (LOC104425496) was used as an internal control, and for each analysis, 3 technical replicates and 3 biological replicates were performed.

### 4.8. Data Analysis

Gene expression levels were calculated using the 2^−ΔΔCT^ method [38,65]. The ΔCt value was the difference between the Ct value of the target gene and the Ct value of the reference gene. The ΔΔCt value was the ΔCt value of the experimental group minus the ΔCt value of the control group. The relative expression level was calculated using the 2^−ΔΔCt^ formula. The statistical analysis was conducted using *t*-tests along with both one-way ANOVA and two-way ANOVA approaches. “NS” represented no significance, “*” represented *p* < 0.05, “**” represented *p* < 0.01, “***” represented *p* < 0.001, “****” *p* < 0.0001. Bar charts were plotted using the ggplot2 (v3.5.1) package in R (v4.3.1).

The *Eucalyptus grandis*-related transcriptome data used in this study were downloaded from the National Genomics Data Center database of the China National Center for Bioinformation, with accession number PRJCA002468 [39]. Quality control was performed using FastQC (version 0.11.9) and MultiQC (version 1.12) [66]. Following raw data preprocessing with Trim Galore (a Cutadapt wrapper, v0.6.10) and Cutadapt (v4.0), genome alignment was performed against the Eucalyptus grandis reference genome using STAR aligner (v2.7.10a) with pre-built genomic indices [67]. The resulting SAM files were converted to BAM format using samtools (v1.16.1), followed by gene-level quantification through FeatureCounts (v1.6.4) with species-specific annotation files [68,69]. Finally, we normalized all samples using R software DEseq2 (v1.42.1) to obtain the final gene expression matrix [70]. Heatmaps were plotted using TBtools Heatmap tools (v2.154).

## 5. Conclusions

Phosphorus is an essential macronutrient for plant development and productivity, and PHRs are major regulators of Pi-starvation signaling. The identification and functional analysis of PHRs in *Eucalyptus* will provide important information for the future design of high-PUE woody plants. In this study, twelve PHRs were characterized in *Eucalyptus grandis*. The gene expression analysis revealed that *EgPHRs* might be involved in the regulation of plant development, JA response, SA response, cold stress, salt stress, and nutrient availability. Notably, the protein–protein interactions and promoter-binding transcription factor analysis suggest that *EgPHRs* might be involved in mitotic chromosome segregation and tissue differentiation in *Eucalyptus*. Overall, our results suggest that *EgPHRs* may play an important role in coordinating plant development, abiotic stresses, and nutrient availability.

## Figures and Tables

**Table 1:** PHR proteins in *Eucalyptus grandis*.

Name	Sequence ID	Size (AA)	MW (KDa)	PI	Instability Index	GRAVY	Subcellular Localization	Group
EgPHR1	LOC104436960	581	63.21	5.98	67.24	−0.80	Nucleus	I
EgPHR2	LOC104450718	498	55.00	5.68	71.40	−0.72	Nucleus	I
EgPHR3	LOC104425247	414	45.41	6.79	42.30	−0.63	Nucleus	II
EgPHR4	LOC104445780	384	43.14	7.62	71.83	−0.76	Nucleus	I
EgPHR5	LOC104442716	378	41.88	6.82	46.29	−0.73	Nucleus	II
EgPHR6	LOC104432550	371	40.93	5.20	53.12	−0.76	Nucleus	I
EgPHR7	LOC104453942	353	39.24	7.14	59.45	−0.75	Nucleus	II
EgPHR8	LOC104451042	344	37.97	7.90	46.69	−0.75	Nucleus	III
EgPHR9	LOC104447696	330	36.09	5.69	53.79	−0.78	Nucleus	I
EgPHR10	LOC104441194	330	35.67	6.39	44.07	−0.70	Nucleus	I
EgPHR11	LOC104456029	316	34.12	6.08	45.25	−0.34	Nucleus	III
EgPHR12	LOC104442369	294	32.13	9.04	50.46	−0.69	Nucleus	III

AA: amino acids, MW: molecular weight, KDa: kilodalton, PI: isoelectric point, GRAVY: grand average of hydropathicity, Group I–III: classification according to the phylogenetic analysis.

## Data Availability

The transcriptome data used in this study were downloaded and reanalyzed from the National Genomics Data Center database (PRJCA002468 for tissue expression, JA response, SA response, salt stress, and boron deficiency).

## Supplementary Materials

The supporting information can be downloaded at: https://www.mdpi.com/article/10.3390/ijms26072958/s1.

## Abbreviations

The following abbreviations are used in this manuscript:

EgPHRs	*Eucalyptus grandis* phosphate starvation responses
Eg	*Eucalyptus grandis*
At	*Arabidopsis thaliana*
Potri	*Populus trichocarpa*
Os	*Oryza sativa*
Chr	Chromosome
FPKM	Fragments per kilobase of transcript per million mapped reads
HMM	Hidden Markov Model
RT-qPCR	Quantitative reverse transcription PCR
WGS	Whole-genome sequencing
PUE	Phosphorus use efficiency
PPI	Protein–protein interaction
SPX	SYG1, Pho81, XPR1
PAP	Purple acid phosphatase
WUS	WUSCHEL
KNOX1	Knotted1-like homeobox 1
WOX13	WUSCHEL-related homeobox 13
SIP1	Smad-interacting protein 1
BSL1	BSU1-like family of Ser/Thr protein phosphatases 1
KCBP1	Kinesin-like calmodulin binding protein 1
