# Supplementary material for: Decoding PHR-Orchestrated Stress Adaptation: A Genome-Wide Integrative Analysis of Transcriptional Regulation Under Abiotic Stress in Eucalyptus grandis
Source: Int J Mol Sci. 2025 Mar 25;26(7):2958. doi: 10.3390/ijms26072958 (PMC11988722; doi:10.3390/ijms26072958)
Supplement: Supplementary file 1 [file ijms-26-02958-s001.zip › Supplementary Figures.pdf]

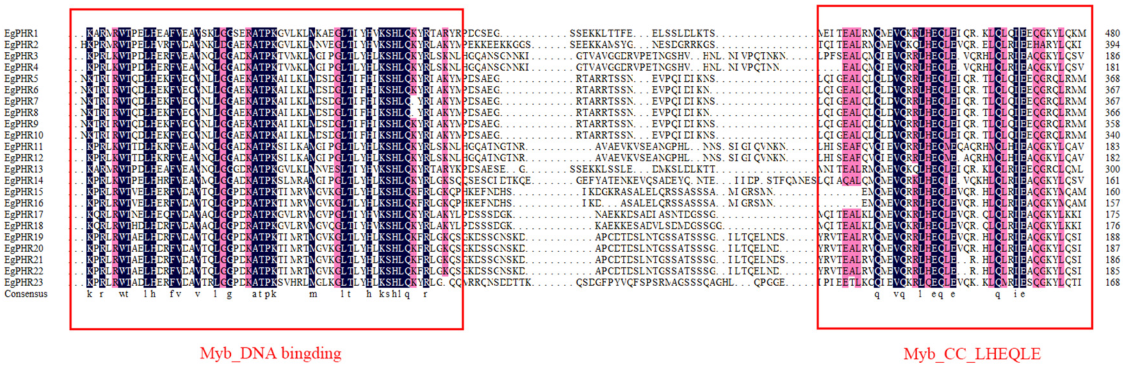


**Figure S1.** Multiple sequence comparison of *Eucalyptus grandis* PHR proteins. The *PHR* genes of *Eucalyptus grandis* all have two conserved structural domains, the Myb_DNA binding domain and the Myb_CC_LHEQLE domain. (The black portion of the sequence represents 95% homology and the purple portion represents 75% homology of the sequence at that site).

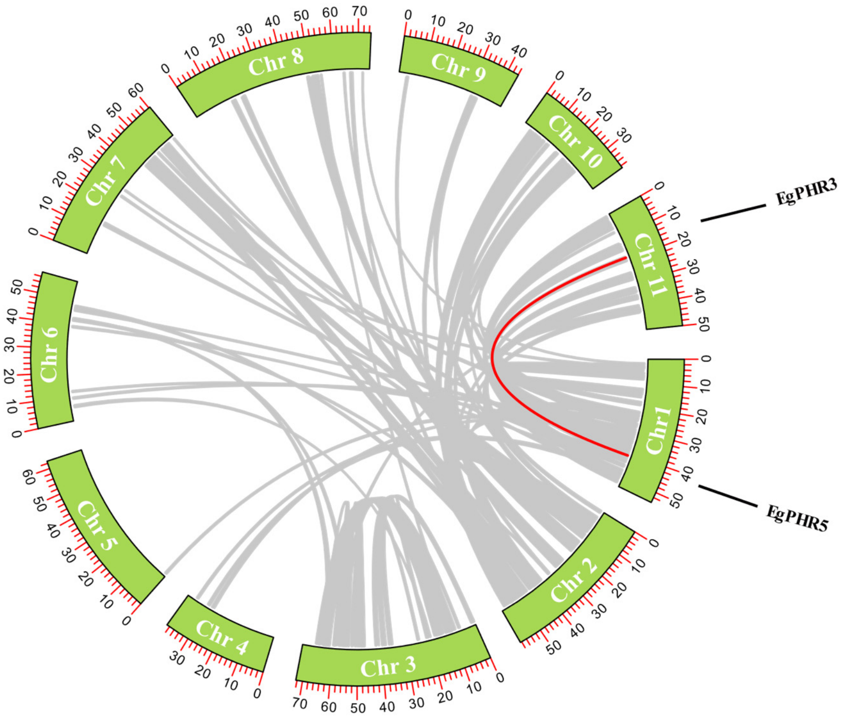


**Figure S2.** Gene duplication and syntenic analysis of *PHR* gene in the *Eucalyptus grandis*. The green parts represented the chromosomes of *Eucalyptus grandis*, the outer scale represented the length of the chromosomes, the circled lines indicated the segmental duplication genes, and the lines marked in red were the segmental duplication pairs within the *EgPHR* genes.

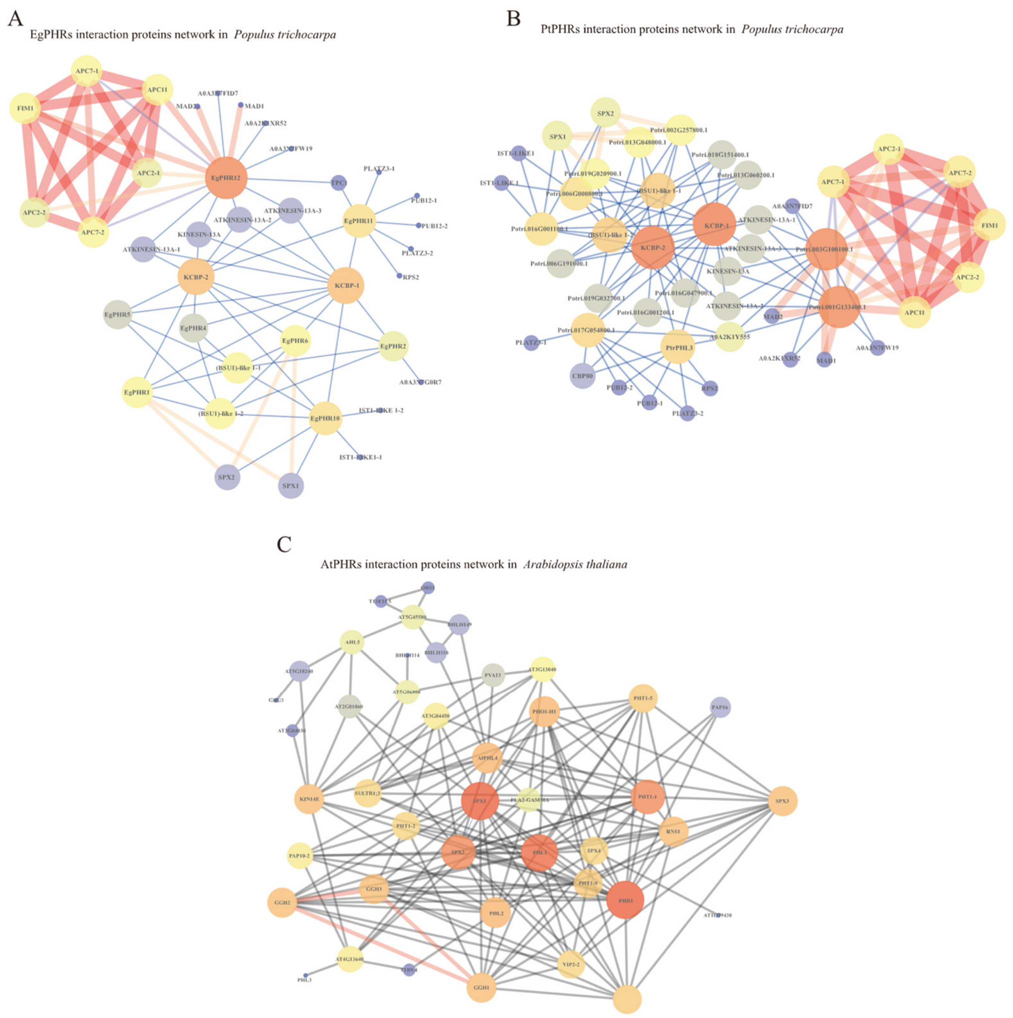


**Figure S3.** Protein interaction networks of EgPHRs, AtPHRs and PrtPHRs. Figure A demonstrated the protein interaction network of EgPHRs in *Populus trichocarpa*; Figure B demonstrated the protein interaction network of PrtPHRs in *Populus trichocarpa*; Figure C demonstrated the protein interaction network of EgPHRs in *Arabidopsis thaliana*.

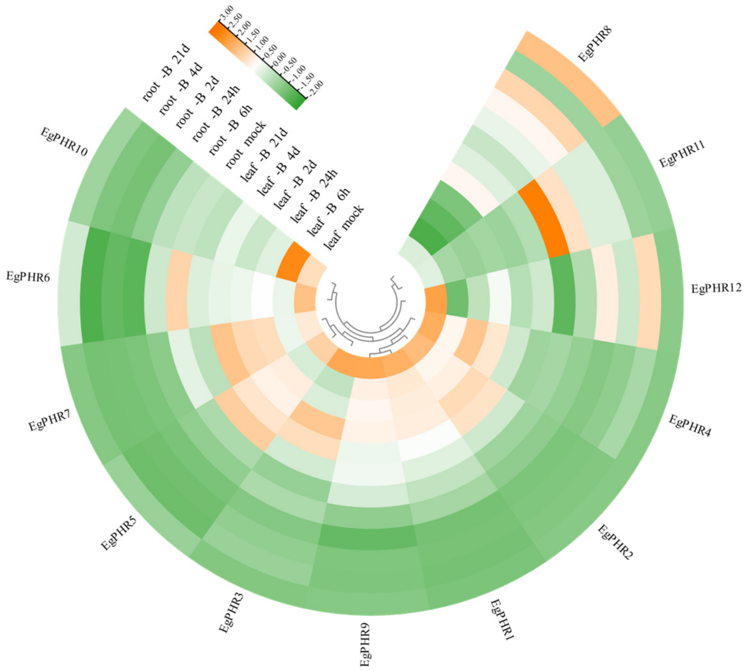


**Figure S4.** Expression of *EgPHR* gene in response to boron deficiency. Normalized all samples using R software DEseq2 to obtain the final gene expression matrix. Heatmaps were plotted using R's pheatmap and ggplot2 packages. Clustering by rows, orange represented high expression and green represented low expression.
